# Landfill leachates and wastewater of maritime origin as possible sources of endocrine disruptors in municipal wastewater

**DOI:** 10.1007/s11356-019-05566-4

**Published:** 2019-07-02

**Authors:** Barbara K. Wilk, Sylwia Fudala-Ksiazek, Małgorzata Szopińska, Aneta Luczkiewicz

**Affiliations:** 1 Department of Water and Wastewater Technology, Faculty of Civil and Environmental Engineering, Gdansk University of Technology, 11/12 Narutowicza St., 80-233 Gdansk, Poland; 2 Department of Sanitary Engineering, Faculty of Civil and Environmental Engineering, Gdansk University of Technology, 11/12 Narutowicza St., 80-233 Gdansk, Poland

**Keywords:** Micropollutants, Landfill leachates, Cruise ship wastewater, Municipal and industrial wastewater, Treatment approach

## Abstract

In this study, wastewater from municipal services, such as a port wastewater reception facility (PRF-WW) and a municipal solid waste plant (MSWP), was tested for the presence of the suspected endocrine-disrupting compounds phthalates (PAEs) and bisphenol A (BPA). PAEs and BPA were found in this study in high concentrations in raw wastewater obtained from passenger ships (RMT-WWs) (up to 738 μg/L and 957 μg/L, respectively) collected in the Port of Gdynia and in landfill leachates (LLs) (up to 536 μg/L and up to 2202 μg/L, respectively) from a MSWP located near Gdynia. In particular, the presence of reprotoxic di(2-ethylhexyl) phthalate (DEHP, up to 536 μg/L in LLs and up to 738 μg/L in RMT-WWs) requires further action because if this compound, as well as other PAEs and BPA, is not degraded by activated sludge microorganisms, it may reach receiving waters and adversely impact aquatic organisms. Therefore, PAEs and BPA should be removed either during the onsite pretreatment of tested industrial wastewater or during tertiary treatment at municipal wastewater treatment plants (WWTPs, representing end-of-pipe technology).

**Electronic supplementary material:**

The online version of this article (10.1007/s11356-019-05566-4) contains supplementary material, which is available to authorized users.

**Figure:**
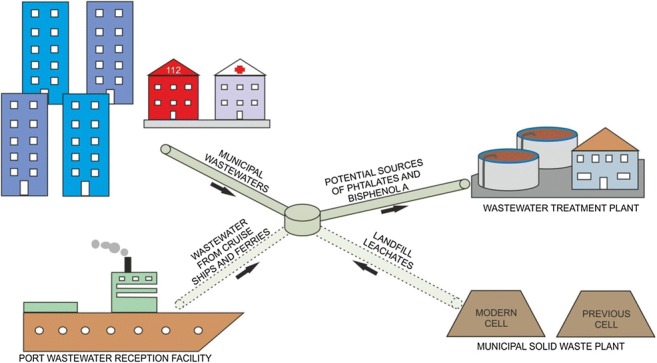
Graphical abstract

## Introduction

Proper wastewater management plays a crucial role in achieving good water status and the potential restoration of water resources. To mitigate the environmental burden of wastewater, the following two approaches are generally considered: cleaner production and end-of-pipe technology (Chour 2001). Cleaner production is thought to reduce resource usage and/or pollution emissions, but past and even current environmental regulations rely far more on the end-of-pipe approach. The quality standards for wastewater discharge, however, can underestimate the impact of a particular substance on the ecosystem due to the limited knowledge of concertation-response effects (Connon et al. 2012).

Micropollutants can enter the aquatic environment through both diffuse and point sources; however, in urbanized regions, wastewater treatment plants (WWTPs) play a crucial role in their dissemination. Conventional WWTPs are effective in macropollutant removal, while micropollutants may pass through the treatment process unchanged or are removed at different rates. Most EU countries are convinced that the presence of micropollutants in the environment poses a serious problem, particularly in highly populated regions, where water resources are used for drinking and irrigation purposes and as wastewater receivers. It has already been confirmed that insufficient removal of micropollutants by WWTPs and by water treatment plants can result in the presence of endocrine-disrupting compounds (EDCs) in drinking water (Albergamo et al. 2019; Badach et al. 2007; Tröger et al. 2018). Of special concern are water bodies that receive a high fraction of treated wastewater discharged from several WWTPs simultaneously because the combined and cumulative impact of micropollutants can occur under such conditions (Logar  et al. 2014). In many countries, reduced dilution potential of surface water bodies occurs during summer droughts, increasingly reported in recent years (Englert et al. 2013).

Thus, there is growing concern about persistent and bioactive micropollutants (Dévier et al. 2011), which may enter the water body via different pathways. In the case of municipal WWTPs, some micropollutants that are resistant to biodegradation are usually not completely removed via conventional WWTP techniques (Luo et al. 2014). Of special concern are priority substances specified by the Directive 2013/39/EU. The current challenge in water policy is, however, not only the monitoring of micropollutants in water bodies but also the identification of their sources and implementation of possible technologies to mitigate their release. Industrial facilities discharge wastewater into the environment either (a) directly through their own sewerage and onsite wastewater treatment system or (b) indirectly via municipal WWTPs. Currently, more industrial plants have decided to pretreat wastewater on site to guarantee the quality required by the relevant legislation (for details, see Table S1 in Supplementary materials). Unfortunately, the effectiveness of micropollutant removal is rarely checked; thus, it is suspected that some emerging compounds (e.g., with limited biodegradation) are still directed to municipal WWTPs (Chour 2001).

Thus, it is necessary to better understand the demands and related costs of complying with the EU standards for water quality (for more, see Table S1). In this study, there was a special focus on wastewater onsite pretreatment of industrial wastewater originating from municipal services, such as landfill leachates (LLs) generated at municipal solid waste plants (MSWPs) and maritime wastewater (MT-WW) from port reception facilities (PRFs-WW). LLs are defined as liquids that pass through deposited solid waste, leaching dissolved and suspended matter, and due to the complex composition of LLs (Fudala-Ksiazek et al. 2016, 2017; Kulikowska and Klimiuk 2008; Renou et al. 2008; Wiszniowski et al. 2006), several processes have been tested to solve the challenging LL treatment issues (Boonnorat et al. 2016; Fudala-Ksiazek et al. 2018; Liu et al. 2017; Mandal et al. 2017; Wojciechowska 2017). The effectiveness of these methods, however, has rarely been analysed in terms of micropollutant removal (Fudala-Ksiazek et al. 2017, 2018; Yi et al. 2017).

The generation of MT-WWs is also of special concern because the number of people transported by cruise liners and ferries on the Baltic Sea has increased by an average of 9.9% annually (from 1.1 million in 2000 to 4.3 million in 2016) (Cruise Baltic 2016, 2017; Kovalevskiene et al. 2017). Furthermore, since January 2013, the Baltic Sea has become the first special area with mandatory limits for the discharge of phosphorus (max 1.0 mg/L or 80% reduction) and nitrogen (max 20 mg/L or 70% reduction) (for details, see Table S1 in Supplementary Materials). These limits make it necessary to equip ships with appropriate treatment systems or to equip ports with wastewater reception facilities (PRFs-WW) (Table S2) (HELCOM 2018). Unfortunately, the presence and dissemination of micropollutants via MT-WWs have been overlooked (Nödler et al. 2014; Carić 2016). If discharged to a PRF-WW, MT-WWs are usually then directed to local WWTPs (Carić 2016; Directive 2455/2001/EC; Prior 2013). However, as in the case of LLs, there is a lack of data regarding the presence of micropollutants in MT-WWs and their fate in municipal wastewater treatment.

The aim of this study is to determine the presence of BPA and selected PAEs (DMP—dimethyl phthalate, DEP—diethyl phthalate, DnBP—di-n-butyl phthalate, BBzP—benzyl butyl phthalate, DEHP—bis(2-ethylhexyl) phthalate, and DnOP—di-n-octyl phthalate) in wastewater generated by MSWP (LLs) and originated from PRF-WW (MT-WW). The selection of BPA and PAEs was related to their common presence in numerous products and tendency to end up in solid waste streams and/or maritime wastewater. Both PAEs and BPA can be ingested by a wide range of marine organisms and then negatively affect their endocrine system, resulting in impaired reproduction, loss of biodiversity, incidence of hormone-sensitive cancers, and other effects (Hermabessiere et al. 2017). According to HELCOM (Baltic Marine Environment Protection Commission, 2010), the concentrations of these compounds in biota from coastal regions are generally high (PAEs: 50 μg kg^−1^ wet weight in fishes and 2500 μg kg^−1^ dry weight in sediment; BPA: 45 μg kg^−1^ wet weight in fishes). In this study, BPA and selected PAEs were also determined in municipal wastewater entering the WWTPs (IN-WWTP). These results are regarded as a baseline condition due to the planned connection of local WWTPs to the abovementioned PRF-WW and MSWP wastewater systems.

## Materials and methods

### Sampling

**Fig. 1:**
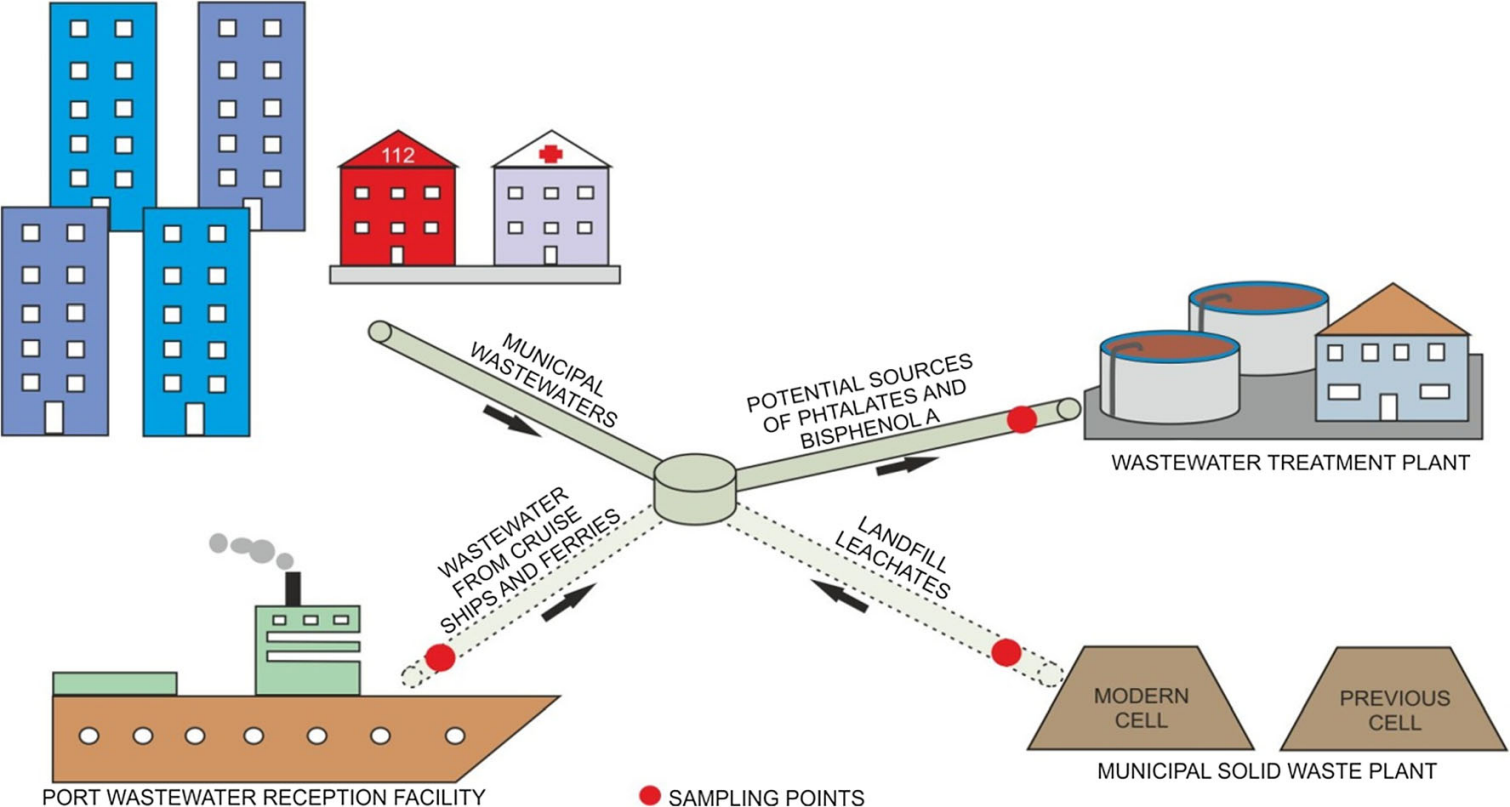
Locations of sampling points: influent of wastewater treatment plant (IN-WWTP); landfill leachates generated by a modern cell (MP-LLs) that meets EU requirements and a previous cell (PP-LLs) with unlimited disposal of biodegradable wastes; wastewater from cruise ships and ferries: raw (RMT-WW) and pretreated onboard (PMT-WW); solid lines represent existing connections and dashed lines represent planned connections between the port reception facilities (PRF-WW) and municipal solid waste plant (MSWP) and the local wastewater treatment plant (WWTP)

In this study, wastewater from a PRF-WW, an MSWP and inflow of a WWTP were tested. The locations of the sampling points are shown in Fig. 1. All samples were collected in pre-cleaned glass amber bottles (1 L) and transported to the laboratory under dark conditions at 4 ± 1 °C.

#### Landfill leachates

In this study, LLs were collected from a modern cell (MP-LLs) and a previous cell (PP-LLs) of a MSWP situated in the Pomerania region (northern Poland). The previous cell, exploited from January 2003 to November 2011, was operated with unlimited disposal of organic wastes, while the disposal of biodegradable wastes in the modern prism (in operation since 2011), due to legislative requirements, is only partly permitted. Along with other EU members, Poland, which landfilled more than 80% of its municipal waste in 1995, was required by Directive 1999/31/EC to progressively reduce the landfilling of biodegradable waste to 75% by 2010, 50% by 2013, and 35% by 2020. Thus, the quality of the MP-LLs is expected to differ significantly from that of the PP-LLs (for details, see Fudala-Ksiazek et al. 2016).

The studied MSWP serves a metropolitan area with a population of approximately 460,000 people and receives ca. 190,000 tons of waste per year, of which 97,000 tons is biodegradable. Samples were collected monthly from January 2015 to April 2016 as 24-h composite samples. In total, 20 samples were collected.

#### Wastewater from cruise ships

The wastewater from cruise ships and ferries was collected in the Port of Gdynia (northern Poland) during the tourist seasons of 2015 and 2016 (from April to October). Samples of raw (RMT-WW) and pretreated (PMT-WW) wastewater were collected from port reception facilities (PRF-WW) during the emptying of the wastewater tanks (the middle stream). In total, 25 wastewater samples were collected, and 15 of the samples had been pretreated in wastewater treatment plants on cruise ships (PMT-WWs). The onboard treatment systems were not reported, just the wastewater status (raw or pretreated). The RMT-WW samples consisted of a mixture of black water (water from toilets) and grey water (other types of wastewaters generated by kitchens, showers, sinks, laundry, and other sources aboard the cruise ships).

#### Municipal wastewater

Samples of municipal wastewater were collected twice a month from January 2015 to December 2016 from the inflow of the WWTP Gdynia-Debogorze (IN-WWTP), northern Poland. In total, 48 samples were collected.

The population equivalent (PE) served by WWTP Gdynia-Debogorze is equal to 440,000 (*Q*_av._ = 55,000 m^3^/day), and its technology consists of mechanical and biological treatment (advanced biological nutrient removal), secondary settling tanks with recirculation of excess sludge, and a chemical system (iron(II) chloride; PIX dosing) for occasional phosphorus removal. Currently, industrial wastewater (mostly from the food industry) contributes 10% of the total wastewater inflow; however, in the near future, the WWTP is planned to receive LLs from the nearby MSWP and wastewater from the PRFs-WW in the Port of Gdynia.

### Chemical analysis

#### Basic physical and chemical analyses

Among the routinely measured parameters, the following parameters were analysed according to the American Public Health Association (APHA 2005): pH and conductivity (by a portable multi-parameter meter, the HL-HQ40d multi, HACH, Germany); inorganic N compounds (N-NH_4_, N-NO_3_, and N-NO_2_), total phosphorus (TP), orthophosphate (P-PO_4_), chemical oxygen demand (COD), chloride (Cl^−^), sulfate (SO_4_^2−^), and sulfides (S^2−^) using a XION 500 spectrophotometer (Dr. Lange, GmbH, Germany); 5-day biochemical oxygen demand (BOD_5_) using the manometric respirometric BOD OxiTop® method; and total suspended solids (TSS) using the gravimetric method.

#### Analysis of phthalates (PAEs) and bisphenol A (BPA)

Selected PAEs (DMP—dimethyl phthalate, DEP—diethyl phthalate, DnBP—di-n-butyl phthalate, BBzP—benzyl butyl phthalate, DEHP—bis(2-ethylhexyl) phthalate, and DnOP—di-n-octyl phthalate) and BPA were determined by gas chromatography mass spectrometry (GC-MS) after prior liquid-liquid extraction to mixture of acetonitrile and tetrahydrofuran in a ratio of 4/1 (*v*/*v*) in the presence of inorganic salts (for details, please see Fig. S1, Supplementary Material). The GC-MS analyses were performed on a semi-polar ZB-5MS column in split mode. The ion energy for electron impact (EI) was 70 eV, and mass detection was performed in the single-ion monitoring (SIM) mode accordingly to Fudala-Ksiazek et al. (2017). The selected ions (*m*/*z*) and retention times used for qualitative and quantitative purposes are shown in Table S2 (Supplementary Material). From 15 samples of PMR-WW, 5 were excluded from micropollutant analyses due to the possible improper onboard management of wastewater treatment plants (see the ‘Results and Discussion’ section). The LLs were also tested for the presence of BPA and PAE: 8 samples of PP-LLs and 9 samples of MP-LLs. Additionally, 6 samples of IN-WWTP were analysed.

#### Quality assurance and quality control (QA/QC)

All data were subjected to precise quality control procedures. External calibration curves (mixture of all selected analytes: DMP, DEP, DnBP, BBzP, DEHP, DnOP, BPA) were used for quantitative analyses. Linearity was checked in the range 10–100 μg/L (*r*^2^ > 0.995 for all analytes). Measurements of samples were performed in triplicate. All sample processing steps of the analytical method were included in the determination of the method detection limit. The method detection limits (LOD) for each analyte were calculated based on the standard deviation of the response (*s*) and the slope of the calibration curve (*b*) according to the formula LOD = 3.3 (*s*/*b*). Method quantification limits (LOQ) were calculated according to the formula LOQ = 3 LOD. The accuracy and precision of the measurements (in %) of individual analytes are presented in Table 1. Blank (acetonitrile and tetrahydrofuran in a ratio of 4/1 (*v*/*v*)) and reference solutions (analyte standard solution concentration—200 μg/L) were run after every ten samples to ensure the precision of the determination of the analytes in each sequence. Moreover, procedure blanks were analysed in the same setup as the samples, using the same reagents, in de-ionized water with levels below the detection limit for each analyte. Moreover, the matrix effect of landfill leachates and wastewaters has been checked through. Selected samples have been spiked by analytes (concentrations were 50 μg/L for DMP, DEP, DnBP, BBzP, DEHP, and DnOP and 735 μg/L for BPA). The variation between spiked samples (with matrix) and the standard solution (without matrix) was less than 10%.

**Table 1:** Basic quality control parameters for GC-MS analysis

Analyte	LOD [μg/L]	LOQ [μg/L]	Recovery^a^ [%]	RSD^a^ [%]
DMP	2.30	7.68	100	6.4
DEP	6.08	20.3	116	8.8
DnBP	16.1	53.6	92.9	7.8
BBzP	0.30	1.00	79.2	12
DEHP	44.8	149	55.6	4.8
DnOP	1.20	4.00	47.4	8.3
BPA	37.5	125	135	7.0

*LOD* limit of detection, *LOQ* limit of quantification, *RSD* relative standard deviation, *DMP* dimethyl phthalate, *DEP* diethyl phthalate, *DnBP* di-n-butyl phthalate, *BBzP* benzyl butyl phthalate, *DEHP* bis(2-ethylhexyl) phthalate, *DnOP* di-n-octyl phthalate, *BPA* bisphenol A

^a^Refers to the standard solution with a concentration of 50 μg/L for each analyte

### Statistical analysis

**Fig. 2:**
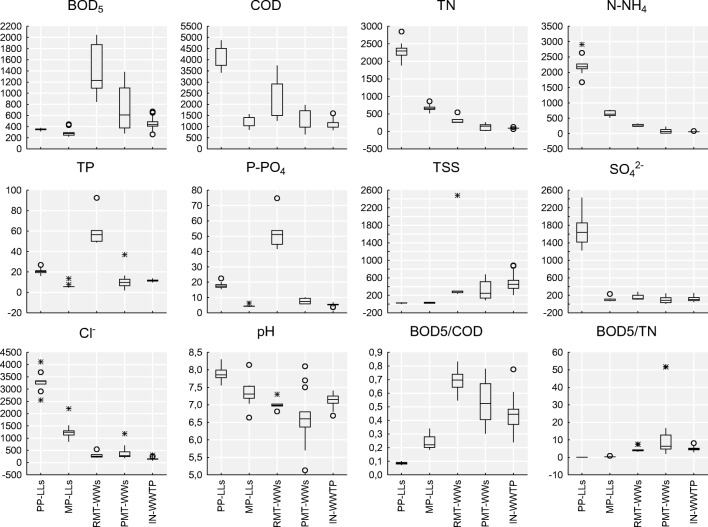
Minimum, maximum, upper quartile, lower quartile, median values, outliers, and extreme values for the parameters of sewage contamination and BOD_5_/COD and BOD_5_/TN for the leachates generated by the previous (PP-LLs) and modern (MP-LLs) cells, raw (RMT-WWs) and pretreated wastewater (PMT-WWs) from cruise ships and ferries, and municipal wastewater entering the WWTP (IN-WWTP)

Data spreadsheets were prepared using Microsoft Excel® 2016 (Microsoft, 2016, USA). Statistical analyses (Fig. 2) were carried out using Statistica 12 (StatSoft, Inc.) software. The distributions of the pollutant concentrations and the basic values of the descriptive statistics are shown using box plots (minimum, maximum, upper quartile, lower quartile, median values, outliers and extreme values). Box plots represent the symmetry or asymmetry of the data well and allow the visualization of the variability among the compared groups. The normality of the data was determined with the Shapiro-Wilk test, while the homogeneity of the variance was assessed with Levene’s test. For all the tests, differences were determined to be statistically significant if *p* < 0.05. The non-parametric Wilcoxon’s signed rank test was used to compare the concentration of contaminants in RMT-WWs and PMT-WWs. The Mann-Whitney *U* test was used to indicate the significance of differences between the values of pollutant concentrations in MP-LLs and PP-LLs.

## Results and discussion

The wastewater that originates from different municipal services is often discharged to municipal wastewater systems, either directly or after onsite pretreatment. Unfortunately, the discharge requirements are limited to the routinely measured parameters used to characterize the wastewater, such as BOD_5_, COD, TSS, TN, and TP (see Table S2 for comparison). Other specific contaminants, such as micropollutants, are not routinely tested. Thus, in this study, the presence of BPA and PAEs, regarded as endocrine-disrupting compounds, was determined in the wastewater generated by municipal services, such as MT-WWs (PMT-WWs and RMT-WWs) from cruise ships calling at the Port of Gdynia and LLs (MP-LLs and PP-LLs) from MSWPs. Due to the planned discharge of LLs and MT-WWs to local municipal wastewater systems, the inflow into the WWTP Gdynia-Debogorze was also analysed (IN-WWTP) to obtain the current baseline conditions (Fig. 1).

### Physico-chemical results

According to the obtained results, the quality of MP-LLs generated by the modern cell (meeting current EU requirements in terms of waste management and disposal) and the quality of PP-LLs generated by the previous cell (exploited without any limits) differ significantly (Table 2; for a detailed description, also see Supplementary Materials).

**Table 2:** Presence of PAEs and BPA in LLs generated by previous (PP-LL) and modern (MP-LL) cells, raw (RMT-WW) and pretreated (PMT-WW) wastewater generated by cruise ships and ferries, and municipal wastewater (IN-WWTP) entering the WWTP

Parameters [μg/L]	Landfill leachates		Cruise ships and ferries^a^		MWTP
	PP-LLs	MP-LLs	RMT-WWs	PMT-WWs	IN-WWTP
DMP	< LOD–27.9^b^ 6/1/1	< LOD–23.1 4/4/1	< LOD–118 3/4/3	< LOD 10/0/0	< LOD 6/0/0
DEP	< LOD, < LOQ 7/1/0	< LOD–46.2 5/2/2	< LOD–43.7 2/4/4	< LOD 10/0/0	< LOD, < LOQ 5/1/0
DnBP	< LOD 8/0/0	< LOD 9/0/0	< LOD 10/0/0	< LOD 10/0/0	< LOD 6/0/0
BBzP	< LOD 8/0/0	< LOD–1.7 8/0/1	< LOD 10/0/0	< LOD 10/0/0	< LOD 6/0/0
DEHP	< LOD–257 4/1/3	< LOD–536 6/2/1	< LOD–738 1/4/5	< LOD 10/0/0	< LOD, < LOQ 3/3/0
DnOP	< LOD 8/0/0	< LOD 9/0/0	< LOQ–52.1 0/6/4	< LOD 10/0/0	< LOD 6/0/0
BPA	856–2202 0/0/8	< LOQ–150 0/6/3	145–957 0/0/10	< LOD 10/0/0	< LOD, < LOQ 2/4/0

*DMP* dimethyl phthalate, *DEP* diethyl phthalate, *DnBP* di-n-butyl phthalate, *BBzP* benzyl butyl phthalate, *DEHP* bis(2-ethylhexyl) phthalate, *DnOP* di-n-octyl phthalate, *BPA* bisphenol A

^a^In this study, 5 out of 15 samples of raw wastewater of maritime transport origin (RMT-WW) were excluded from micropollutant analyses due to the possible improper onboard management of wastewater treatment plants

^b^The first line is the range of micropollutant concentrations. The second line (< LOD/< LOQ/> LOQ) is the number of samples with the results below the LOD (< LOD), between the LOD and the LOQ, and greater than the LOQ (> LOQ), respectively (for details, see Table 1)

The Mann-Whitney *U* test revealed that there was a significant difference (*p* < 0.05) in the mean pollutant concentrations between the MP-LLs and the PP-LLs. Higher pollutant concentrations were found in the PP-LLs, except for BOD_5_ and TSS values, due to the generation of the methane phase (Fudala-Ksiazek et al. 2016, 2017). This finding was confirmed indirectly by the stable methane production (personal communication, exploiter at studied MSWP), low BOD_5_/COD ratio (0.090 ± 0.008), and COD and BOD_5_ values (4120 ± 11 mg O_2_/L and 351 ± 21 mg O_2_/L, respectively), with a small coefficient of variation (*V*_COD_ = 11.3%, *V*_BOD5_ = 5.9%). In the case of MP-LLs, the following average values of COD were determined: 1248 ± 236 mg O_2_/L (*V*_COD_ = 18.9%) and BOD_5_ 297 ± 76 mg O_2_/L (*V*_BOD5_ = 25.6%). The average BOD_5_/COD ratio in MP-LLs was equal to 0.33 ± 0.24, indicating the presence of readily biodegradable compounds in a total pool of organic matter.

Phosphorus in both the PP-LLs and MP-LLs occurred mainly as P-PO_4_. In the MP-LLs, P-PO_4_ accounted for 84.9% ± 4.0% of the TP, while in the PP-LLs, it accounted for 69.0% ± 13.0% of the TP (Fig. 2). The TN in the PP-LLs reached 2296 ± 264 mg N/L (*V*_TN_ = 11.5%), while the TN in MP-LLs was much lower, with a value of 670 ± 97 mg N/L (*V*_TN_ = 14.5%). In both cells, nitrogen was released mainly in the mineral form of ammonia and was equal to 2231 ± 340 mg N-NH_4_/L (*V*_N-NH4_ = 15.2%) in the PP-LLs and 649 ± 93 mg N-NH_4_/L (*V*_N-NH4_ = 14.4%) in the MP-LLs. The significantly higher nitrogen content in the PP-LLs than in the MP-LLs can again be explained by the unlimited disposal of biodegradable waste during the period of the previous cell exploitation.

Typically, in LLs, high conductivity and the presence of chloride and sulfate ions are also usually noted (Fan et al. 2006; Kawai et al. 2012), which was confirmed by the present study.

The pollution load of the LLs was significant, even when compared with the raw wastewater generated by cruise ships (RMT-WWs), and both were much more concentrated than municipal wastewater (IN-WWTP). The high pollution load in the RMT-WWs may be a result of the limited water volume used onboard (Fig. 2). In this study, the tested RMT-WW samples were a mixture of black water and grey water, and their parameters varied in wide ranges, e.g., TSS from 234 to 2483 ± 885 mg/L, COD from 1260 to 3744 ± 862 mg O_2_/L, and BOD from 845 to 2045 ± 409 mg O_2_/L (Fig. 2). Other authors also reported a high variability in raw wastewater generated by cruise ships (Prior 2013; King County Wastewater Treatment Division 2007). This variability is mainly caused by the different onboard services offered (e.g., restaurants, spas, swimming pools, and bars), cruise duration, and, within some assumed limits, the number of passengers on board. However, due to the low COD/BOD_5_ ratio (< 1.8), RMT-WWs can be regarded as susceptible to biological degradation. The high nitrogen and phosphorus concentrations are noteworthy (TN up to 544 mg N/L and TP up to 92.4 mg P/L) in RMT-WWs, with average high proportions of ammonia (84.0%) and phosphates (87.0%). The obtained values of N-NH_4_ (226–332 mg/L) and P-PO_4_ (41.7–74.8 mg/L) were much higher in RMT-WWs than the values typically observed in municipal sewage (Heinrich and Kozak 2009). The presence of phosphorus is most likely a result of the considerable amount of cleaning agents used in different services offered by cruise ships, as confirmed by Wilewska-Bien et al. (2018). Additionally, the spa facilities often offer brine baths and use chemical water softeners, and in some ships, seawater is used for flushing the toilets, which can explain the high concentrations of sulfate and chloride (162 ± 62 mg SO_4_^2−^/L, 299 ± 107 mg Cl^−^/L) noted in the RMT-WWs in this study. Moreover, TSS especially varied across a wide range in the RMT-WWs, from 234 to 2488 mg/L, but values up to 9660 mg TSS/L have also been reported (Mróz 2017; Sun et al. 2010a, 2010b). The average pH value of raw sewage was 7.00 ± 0.13, and the variability of the obtained values in the tested samples was small (*V*_pH_ = 1.9%).

Because the Baltic Sea is particularly susceptible to eutrophication and is regarded by MARPOL as a special area for wastewater discharge (Cruise Baltic 2017; Kovalevskiene et al. 2017; Perić et al. 2016), special attention should be paid to limit the excess inflow of biogenic substances into the sea (Pihlajamäki and Tynkkynen 2011). According to the obtained results, the tested RMT-WWs indicated the possibility for nitrogen (315 ± 112 mg TN/L, *V*_TN_ = 31.0%) removal via biological methods because the average BOD_5_/TN ratio (4.0 ± 0.4 g BOD_5_/g TN) was higher than the level necessary to support denitrification with organic carbon (3.5 g BOD_5_/g TN) (Swinarski et al. 2009) (Fig. 2). Thus, onboard wastewater treatment is usually based on biological degradation (Sun et al. 2010a, 2010b) and membrane separation (membrane bioreactor system, MBR); these treatments meet the MARPOL Convention standards for phosphorus and nitrogen removal (MEPC.227(64) 2012). Interestingly, in some cases, the quality of cruise ship wastewater that was reported as pretreated onboard (PMT-WW) differed considerably from the limits required by the MARPOL Convention (Fig. 2). Unsatisfactory adherence to the levels of the routinely measured parameters by onboard wastewater treatment facilities is surprising because, according to a personal communication (Technical Ship Management 2019, Sp. z o.o.), most of the cruise ships are equipped with advanced MBR systems. For instance, in some of the PTM-WW samples tested in this study, parameters such as COD (647–1970 mg O_2_/L), BOD_5_ (280–1384 mg O_2_/L), TN (22.7–261 mg/L), N-NH_4_ (2.8–228 mg/L), TP (1.9–36.9 mg/L), P-PO_4_ (5.6–10.1), and Cl^−^ (190–1184 mg/L) were equal to or greater than those of the raw municipal wastewater (IN-WWTP) (Fig. 2). Thus, the quality of the wastewater declared by ship owners as pretreated (PMT-WW) was questionable in many cases (Fig. 2), suggesting improper management of onboard wastewater treatment plants.

### Micropollutants

For the tested endocrine disruptors, the LLs were expected to be the most important source of PAEs and BPA among the analysed samples because both substances are used in numerous products that generally end up in wastewater or solid waste streams. However, according to the obtained results, raw wastewater generated by cruise ships (RMT-WWs) was significantly more polluted with PAEs and BPA than the LLs.

In the case of LLs, a significant difference in PAE and BPA concentrations was observed between the MP-LLs and the PP-LLs undoubtedly caused by the limitations imposed by the EU regulations (Directive 2018/850/EU amending Directive 1999/31/EC) on the deposition of biodegradable and recycled materials in modern cells (MP-LLs) (Table 2 and Table S3 in Supplementary Materials). Thus, the level of BPA in the MP-LLs varied from below the limit of quantification (< LOQ) up to 150 μg/L, while in the PP-LLs, it reached 2202 μg/L. High BPA concentrations in municipal LLs were also noted by others and ranged from 26 to 8400 μg/L (Urase and Miyashita 2003; Morin et al. 2015; Teuten et al. 2009).

In LLs, the presence of PAEs and BPA is mainly connected with their release from parent products (especially low molecular weight phthalates such as DMP and DEP) and with biodegradation. In the study by Schwarzbauer et al. (2002), the observed BPA values were 4,200–25,000 μg/L, while a Norwegian survey reported BPA concentrations in the range of 1–62 μg/L (Arp et al. 2017). On the other hand, Kurata et al. (2008), who examined the leachate from 38 landfills in Japan, determined the maximum BPA value to be 3600 μg/L. The results presented also show that BPA is potentially one of the most frequent micropollutants found in LLs. It should be noted that the BPA values in LLs depend on many factors, such as landfill cell age, the type of waste deposited, and the way the landfill site is operated (Kulikowska 2009).

In the case of PAEs, the highest maximum concentrations in the LLs were associated with DEHP, DMP, and DEP, with values up to 536 μg/L, 27.9 μg/L, and 46.2 μg/L, respectively. According to this and other studies, DEHP, which was classified as a Category 1B reprotoxin by the European Union’s REACH legislation, is the most frequently observed PAE in LLs (Asakura et al. 2004; Zhang and Wang 2009). Wowkonowicz and Kijeńska (2017) detected DEHP < 1.3 to 73.9 μg/L and DMP < 0.6 to 1.98 μg/L to 4.72 μg/L in LLs generated by an old cell located in central Poland. Kalmykova et al. (2013) reported that DEHP concentrations in LL samples ranged from < 1.0 to 23 μg/L and that DEP concentrations ranged from < 0.10 to 22 μg/L, while DnOP and DnBP were not detected.

In the case of MT-WW, Westhof et al. (2016) reported that PAEs and BPA occur mainly in grey water, while pharmaceuticals are predominant in black wastewater (Nödler et al. 2014). In this study, the presence of 4 out of the 6 analysed PAEs was noted in RMT-WWs discharged from ships (Table 2). DnBP and BBzP were not detected in the analysed samples (LOD < 16.1 μg/L and LOD < 0.3 μg/L, respectively; for details, see Table 1 and Table 2), while BPA, DEHP, DMP, DnOP, and DEP were detected at maximum concentrations of 957 μg/L, 738 μg/L, 118 μg/L, 52.1 μg/L, and 43.7 μg/L, respectively (Table 2). High concentrations of BPA and PAEs in RMT-WWs can be explained by their abundance in products that are in everyday use onboard the ship (e.g., plastic bottles and containers). The ship restaurants and services also utilize and process food in synthetic packaging, which increases direct (ingestion) and indirect (respiratory system and dermal exposure) consumption and excretion of BPA and PAEs (Westhof et al. 2016; Crain et al. 2007). Among PAEs, the highest concentration in RMT-WWs was detected for DEHP and DMP. DMP is often present in non-plastic products such as pharmaceuticals and personal care products (Bui et al. 2016; Larsson et al. 2017). DMP is of concern because the growing demand for beauty services has caused even smaller and older cruise ships to offer the highest-quality beauty salons (hair/barber and nail stylists) and spa services (aqua-spas, body wraps, mud baths, body and facial massages, etc.). All of the abovementioned facilities utilize products that contain both PAEs and BPA.

On the other hand, DEHP, BBzP, and DnBP, which were confirmed to cause the so-called phthalate syndrome in animals (e.g., cryptorchidism, hypospadias, and shortened anogenital distance) (Foster et al. 2000; Gray Jr et al. 2000; Mylchreest et al. 2000) and are suspected to similarly affect humans (Suzuki et al. 2011; Swan et al. 2005), have already been prohibited in toys, childcare articles, and cosmetic products (EC No 1223/2009; Directive 2005/84/EC). Thus, the noticeable presence of DEHP in current RMT-WWs is surprising and requires future study.

Fortunately, both PAEs and BPA were below detection limits (< LOD) in PMT-WWs, which may confirm that the onboard MBR systems are effective in removing these tested compounds. In the literature (Judd 2008; Karim and Mark 2017; Mitra et al. 2016), however, the opinion regarding the effectiveness of MBR reactors in micropollutant removal varies, which is probably a result of the membrane system used in MBR (micro/ultrafiltration) and/or the compounds under consideration. Nevertheless, further research is required because knowledge on the fate of micropollutants generated by maritime transport is limited.

**Fig. 3:**
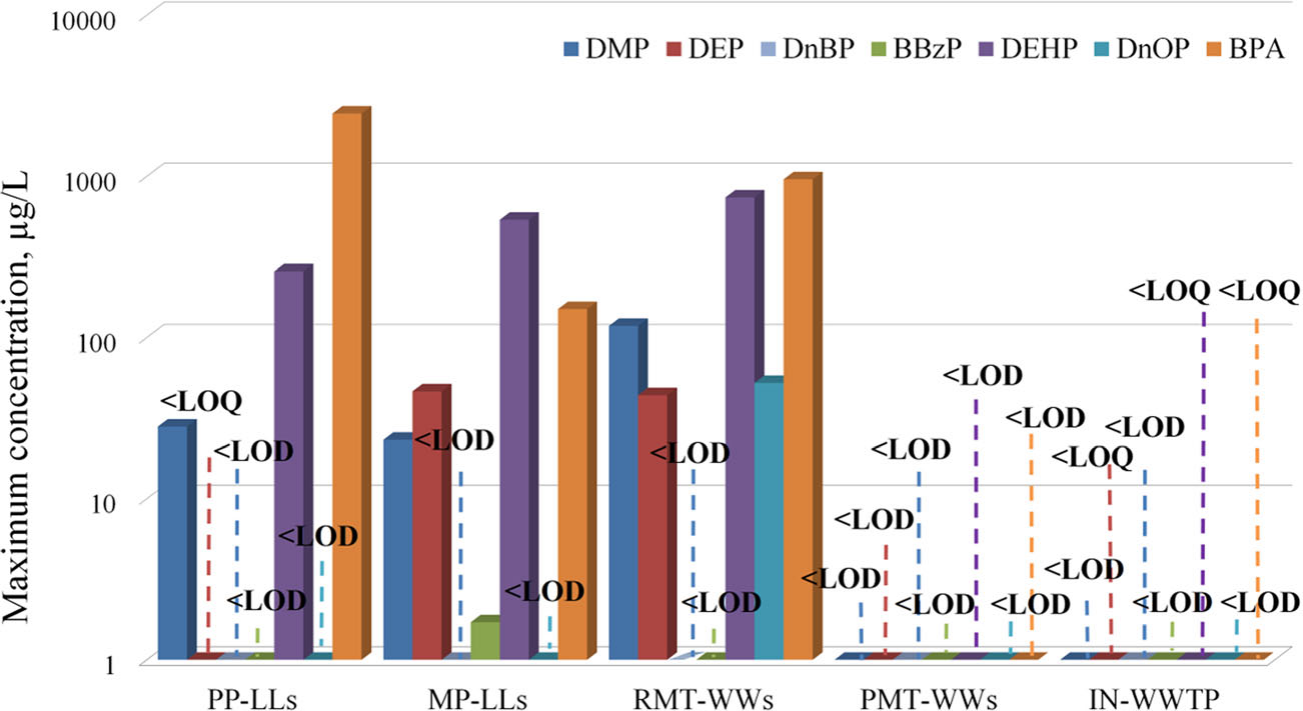
Maximum concentrations of PAEs (phthalates: DMP—dimethyl phthalate, DEP—diethyl phthalate, DnBP—di-n-butyl phthalate, BBzP—benzyl butyl phthalate, DEHP—bis(2-ethylhexyl) phthalate, and DnOP—di-n-octyl phthalate) and BPA (bisphenol A) in landfill leachates generated by previous (PP-LL) and modern (MP-LL) cells, raw (RMT-WW) and pretreated (PMT-WW) wastewater generated by cruise ships and ferries, and municipal wastewater (IN-WWTP) entering the WWTP

In this study, PAEs and BPA were also tested in the wastewater currently entering the local WWTP (IN-WWTP) due to the planned connection of municipal wastewater systems with wastewater streams generated at the local MSWP and collected by PRF-WW. The obtained results did not reveal the presence of PAEs and BPA in the IN-WWTP in quantifiable concentrations (Fig. 3).

In the context of industrial wastewater treatment, the presence of contaminants discharged into the municipal wastewater system that may pass untreated through the WWTPs and have a negative impact on the receiving waters should be considered. For this reason, in the current study, proper approaches to the management of industrial wastewaters originating from municipal services (LLs and in MT-WWs) and the municipal wastewater system are crucial (cleaner production and end-of-pipe technology). Every year, approximately 2700 m^3^ of leachate is generated by the studied MSWP and is currently directed to the onsite pretreatment reverse osmosis (RO) installation. However, although RO plants can be effective at removing micropollutants from LLs, this technology unfortunately also generates many operational difficulties. Major problems also occur with concentrate management (Fudala-Ksiazek et al. 2016, 2017, 2018) and during intense rainfall events (mainly in spring and early autumn), when a large LL stream is directed to the municipal wastewater system without pretreatment, due to the limited flow that the RO plant can handle. In addition, the discharge of MT-WW generated by cruise ships and ferries to the Port of Gdynia reception facilities is estimated to exceed 4598.9 m^3^ per year (Mróz 2017). However, while wastewater pretreated in onboard MBR installations (PMT-WWs) was free of PAEs and BPA, their concentrations in raw wastewater (RMT-WWs) were generally higher than those in the LLs tested in this study. Thus, taking into consideration the environmental sustainability of receiving waters and current baseline conditions (lack of PAEs and BPA in IN-WWTP), a strategic perspective in municipal wastewater management is needed.

Such efforts are of high importance because BPA and PAE removal in activated sludge systems varied widely from 81 to 99% for BPA and 20 to 93% for PAEs, with biodegradation and sorption strongly influenced by operational conditions (hydraulic retention time, solids retention time, wastewater temperature, etc.) (Besha et al. 2017; Grandclement et al. 2017; Hale 2003; Luo et al. 2014; Roslev et al. 2007). For this reason, endocrine disruptors can be discharged via treated wastewater in concentrations of environmental significance.

## Conclusion

The presence of micropollutants in the wastewater generated by MSWPs (LLs) and maritime transport (MT-WWs) is crucial if these compounds exhibit limited biodegradability and, after discharge to municipal WWTP, pass untreated as inert organic matter. This situation is of special concern because the final receiver of WWTP effluent is the Baltic Sea coastal area, which, among other uses, is popular for tourism and recreation as well as for professional and recreational fisheries. Based on the obtained results, it can be concluded that the routinely measured parameters of both LLs and RMT-WWs (mixture of raw grey and black sewage) are higher than assumed for municipal wastewater entering a WWTP (IN-WWTP). These streams are also highly polluted with BPA and PAEs. Special attention should be given to the classified reprotoxin DEHP, which was present in elevated concentrations of up to 257 μg/L in PP-LLs, 536 μg/L in MP-LLs, and 738 μg/L in RMT-WWs. Fortunately, in PMT-WWs and IN-WWTP, both PAEs and BPA were below detection/quantification limits (< LOD/< LOQ). Nonetheless, questions arise regarding sustainable ways to ensure good water status in the future if the MSWP wastewater system and the port reception facility system are to be combined with the municipal wastewater system. Thus, it is essential first to monitor the particular wastewater system to obtain an accurate understanding of the existing impacts that wastewater may have on the sensitive area of the Baltic Sea due to the presence of endocrine disruptors and other micropollutants of concern. Having this knowledge and taking into account local conditions and preferences, the most effective approach can be chosen between onsite pretreatment or end-of-pipe technology.

## Electronic Supplementary Material

ESM 1 (DOCX 100 kb)

## Abbreviations

BPA: Bisphenol A
BBzP: Butyl benzyl phthalate
DEP: Diethyl phthalate
DnBP: di-n-Butyl phthalate
DEHP: di(2-Ethylhexyl) phthalate
DMP: Dimethyl phthalate
DnOP: Diisodecyl phthalate
IN-WWTP: Influent of wastewater treatment plant
LLs: Landfill leachates
MP-LLs: Landfill leachates generated by modern cells that meet EU requirements
MSWP: Municipal solid waste plant
MT-WW: Maritime wastewater
PAEs: Phthalates
PP-LLs: Landfill leachates generated by a previous cell operated with unlimited disposal of biodegradable wastes
PRF-WW: Port reception facilities
PMT-WW: Pretreated (onboard) maritime wastewater from a cruise ship
RMT-WW: Raw wastewater from a cruise ship (mixture of grey and black water)
WWTP: Wastewater treatment plant
