# Supplementary material for: Landfill leachates and wastewater of maritime origin as possible sources of endocrine disruptors in municipal wastewater
Source: Environ Sci Pollut Res Int. 2019 Jul 2;26(25):25690–701. doi: 10.1007/s11356-019-05566-4 (PMC6719331; doi:10.1007/s11356-019-05566-4)
Supplement: Supplementary file 1 — (DOCX 100 kb) [file 11356_2019_5566_MOESM1_ESM.docx]

**Supplementary Material for**

**Landfill leachates and wastewater of maritime origin as possible sources of endocrine disruptors in municipal wastewater**

Barbara K. Wilk ^a*^, Sylwia Fudala-Ksiazek ^b^, Małgorzata Szopińska ^a^, Aneta Luczkiewicz ^a*^

^a^ Gdansk University of Technology, Faculty of Civil and Environmental Engineering, Department of Water and Wastewater Technology, 11/12 Narutowicza St., Gdansk 80-233, Poland; [barbara.k.wilk@pg.edu.pl](mailto:barbara.k.wilk@pg.edu.pl), [ansob@pg.gda.pl](mailto:ansob@pg.gda.pl), malgorzata.szopinska@pg.edu.pl

^b^ Gdansk University of Technology, Faculty of Civil and Environmental Engineering, Department of Sanitary Engineering, 11/12 Narutowicza St., Gdansk 80-233, Poland; sksiazek@pg.edu.pl

^*^Corresponding authors: e-mail: [barbara.k.wilk@pg.edu.pl](mailto:barbara.k.wilk@pg.edu.pl), ansob@pg.gda.pl

**Table of content:**

S1. Detailed characteristic of landfill leachates obtained from modern and previous cell………1

S2. Regulations regarding the direct discharge of maritime wastewater into the Baltic Sea……3

*Table S1. The limits for industrial wastewater discharge in to the municipal wastewater system (requirements of Urban Waste Water Treatment Directive for discharge from WWTP with more than 100 000 PE) and for the direct discharge of maritime wastewater into the Baltic Sea sensitive area (MEPC.227(64)).*

S3. Analytical procedure supporting details…………………………………………………. ..4

*Figure S1. Detailed sample preparation procedure for GC-MS analysis*

*Table S2. Basic parameters of GC-MS analyses.*

S4. Detailed data analysis results……………………………………………………………….5

*Table S3. Presence of PAEs and BPA in LLs generated by previous (PP-LLs) and modern (MP-LLs) cells, raw (RMT-WWs) and pretreated (PMT-WWs) wastewater generated by cruise ships and ferries and municipal wastewater (IN-WWTP) entering WWTP.*

**S1. Detailed characteristic of landfill leachates obtained from modern and previous cell**

According to the obtained results, the quality of MP-LLs generated by the modern cell (meeting current EU requirements in terms of waste management and disposal) and quality of PP-LLs generated by previous cell (exploited without any limits) differ significantly (Table 2).

Mann-Whitney U Test revealed that there was significant difference (p < 0.05) between the means pollutant concentrations in MP-LLs and PP-LLs. Higher pollutant concentrations were found in PP-LLs, except for BOD_5_ and TSS values, due to the already obtained methane phase (Fudala-Ksiazek et al., 2016, 2017).

In PP-LLs low BOD_5_/COD ratio (0.090±0.008) and COD and BOD_5_ values with a small coefficient of variation (V_COD_ = 11.3%, V_BOD5_ = 5.9%) indirectly confirms the stable methane production in this prisms (personal communication, exploiter at studied MSWP). In MP-LLs the average BOD_5_/COD ratio in MP-LLs was equal to 0.33±0.24, indicating the presence of readily biodegradable compounds in a total pool of organic matter. With time it can be expected, however, that the amount of BOD_5_ will decrease in MP-LLs, as observed for PP-LLs. Additionally lower COD and BOD values noted in MP-LLs, when compared with PP-LLs, can be additionally explained by the much lower share of biodegradable waste in the waste stream that is currently deposited due to the new EU waste policy (Directive 2018/850/EU amending Directive 1999/31/EC). The significantly higher nitrogen content in P-LLs (up to 2,296±264 mgN/L) than in MP-LLs (up to - 670±97 mgN/L) can be also explained by the unlimited disposal of biodegradable waste during the period of the previous cell exploitation

Moreover, the samples collected during this study had the following pH values: 7.3±0.4 (V_pH_ = 5.3%) in MP-LLs and 7.9 ±0.2 (V_pH_ = 2.6%) in PP-LLs. The pH of LLs ranges usually from 5.8 to 8.5 (Christensen et al., 2001; Kulikowska, 2009; Moody & Townsend, 2017), and the alkaline PP-LLs confirmed the mature stage of this cell, as observed by others (Jorstad et al., 2004; Naveen et al., 2016).

Typically, in the LLs, high conductivity and the presence of chloride and sulfate ions are also usually noted (Fan et al, 2006; Kawai et al, 2012) which was confirmed by the present study. In PP-LLs, 1,640±398 mgSO_4_^2-^/L, (V_SO4_^2-^ = 24.3%) and 3,297±415 mgCl^-^/L (V_Cl_^-^=12.6%) were detected, while in MP-LLs, both ions were present in lower concentrations (107±48 mgSO_4_^2-^/L; V_SO4_^2-^ = 45.0% and 1,294±365 mgCl^-^/L; V_Cl_^-^ = 28.2%). One of the factors that clearly impacted these concentrations, especially that of Cl^-^ in MP-LLs and PP-LLs, was the management of retentate/concentrate from the reverse osmosis plant, which was recycled to the landfill cells (please compare with Fudala-Ksiazek et al. 2016, 2017).

**Literature:**

Christensen, T. H.; Kjeldsen, P.; Bjerg, P. L.; Jensen, D. L.; Christensen, J. B.; Baun, A.; Albrechtsen, H.-J.; Heron, G. Biogeochemistry of landfill leachate plumes. Appl. Geochem. 2001, 16 (7-8), 659-718; doi 10.1016/s0883-2927(00)00082-2.

Fan, H.-j.; Shu, H.-Y.; Yang, H.-S.; Chen, W.-C. Characteristics of landfill leachates in central Taiwan. Sci. Total Environ. 2006, 361 (1-3), 25-37; doi 10.1016/j.scitotenv.2005.09.033.

Fudala-Ksiazek, S.; Pierpaoli, M.; Kulbat, E.; Luczkiewicz, A. A modern solid waste management strategy – the generation of new by-products. Waste Manage. 2016, 49, 516-529; doi 10.1016/j.wasman.2016.01.022.

Fudala-Ksiazek, S.; Pierpaoli, M.; Luczkiewicz, A. Fate and significance of phthalates and bisphenol A in liquid by-products generated during municipal solid waste mechanical-biological pre-treatment and disposal. Waste Manage. 2017, 64, 28-38; doi 10.1016/j.wasman.2017.03.040.

Kawai, M.; Purwanti, I. F.; Nagao, N.; Slamet, A.; Hermana, J.; Toda, T. Seasonal variation in chemical properties and degradability by anaerobic digestion of landfill leachate at Benowo in Surabaya, Indonesia. J. Environ. Manage. 2012, 110, 267-275.

Kulikowska, D. Charakterystyka oraz metody usuwaina zanieczyszczeń zanieczyszczeń organicznych z odcieków pochodzących z ustabilizowanych składowisk odpadów komunalnych. Ecol. Chem. Eng. S 2009, 16 (3), 389-402.

Moody, C. M.; Townsend, T. G. A comparison of landfill leachates based on waste composition. Waste Manage. 2017, 63, 267-274; doi 10.1016/j.wasman.2016.09.020.

Jorstad LB, Jankowski J, Acworth RI (2004) Analysis of the distribution of inorganic constituents in a landfill leachate-contaminated aquifer: Astrolabe Park, Sydney, Australia. Environ Geol 46(2). https://doi.org/10.1007/s00254-004-0978-3

Naveen BP, Mahapatra DM, Sitharam TG, Sivapullaiah PV, Ramachandra TV (2016) Physico-chemical and biological characterization of urban municipal landfill leachate. Environ Pollut 220:1–12. https://doi.org/10.1016/j.envpol.2016.09.002

**S2. Regulations regarding the direct discharge of maritime wastewater into the Baltic Sea**

**Table S1.** The limits for industrial wastewater discharge in to the municipal wastewater system (requirements of Urban Waste Water Treatment Directive for discharge from WWTP with more than 100 000 PE) and for the direct discharge of maritime wastewater into the Baltic Sea sensitive area (MEPC.227(64)).

| Parameters/ sewage discharge | Unit | Baltic Sea | Municipal Wastewater System |
| --- | --- | --- | --- |
| COD | mg/L | 125 Qi/Qe^1^, MGM | ** |
| BOD_5_ | mg/L | 25 Qi/Qe  without nitrification, MGM | ** |
| TSS | mg/L | 35 Qi/Qe, MGM | ** |
| pH | _ | 6 - 8.5, MIV-MV | 6.5 – 12.5 |
| Thermotolerant Coliform | MPN/100 ml | 100, MGM | _ |
| Total Residual Chlorine | mg/L | 0.5, MV | _ |
| Cl^-^ | mg/L | _ | 1000 |
| Total Nitrogen | mg/L | 20 Qi/Qe (or at least 70%  reduction), MGM | ** |
| N-NH_4_ | _ | _ | 100 – 200 |
| Total Phosphorus | mg/L | 1, 0 Qi/Qe (or at least 80% reduction), MGM | ** |
| Conductivity | mS/cm | _ | ** |

^1^Dilution Factor (Qi/Qe) of 1

MGM- Maximum Geometric Mean During Test Period, MIV - Minimum Value During Test Period,
MV-Maximum Value During Test Period.

**Literature:**

MEPC.227(64), The Marine Environment Protection Committee (2012) Guidelines on implementation of effluent standards and performance tests for sewage treatment plants. Resolution, Annex 22

**S3. Analytical procedure supporting details**


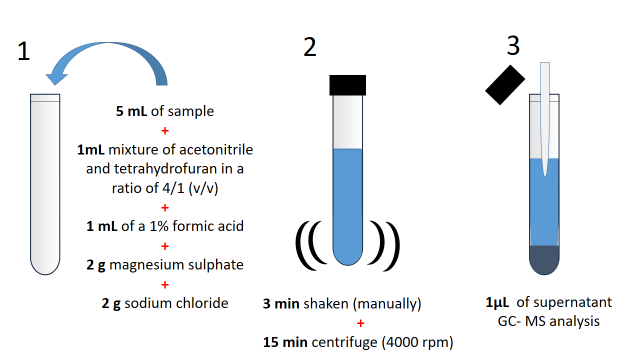


Figure S1. Detailed sample preparation procedure for GC-MS analysis

**Table S2.** Basic parameters of GC-MS analyses.

| **Analyte^1^** |  | **Retention time [min]** | **Quantifier [m/z]** | **Qualifier [m/z]** |
| --- | --- | --- | --- | --- |
| **DMP** |  | 5.64 | 163 | 77 |
| **DEP** |  | 6.10 | 149 | 177 |
| **DnBP** |  | 7.16 | 149 | 76 |
| **BBzP** |  | 8.29 | 149 | 91 |
| **DEHP** |  | 8.62 | 167 | 149 |
| **DnOP** |  | 9.26 | 149 | 279 |
| **BPA** |  | 7.80 | 213 | 228 |

^1^ Abbreviations: DMP - dimethyl phthalate, DEP - diethyl phthalate, DnBP - di-n-butyl phthalate, BBzP - benzyl butyl phthalate, DEHP - bis(2-ethylhexyl) phthalate, DnOP - di-n-octyl phthalate; BPA – bisphenol A

S4. Detailed data analysis results

Table S3. Presence of PAEs and BPA in landfill leachates generated by previous (PP-LLs) and modern (MP-LLs) cells, raw (RMT-WWs) and pretreated (PMT-WWs) wastewater generated by cruise ships and ferries and municipal wastewater (IN-WWTP) entering WWTP.

| Date | DMP | DEP | DnBP | BBzP | DEHP | DnOP | BPA |
| --- | --- | --- | --- | --- | --- | --- | --- |
| 01.2015 | 27.9 | < LOD | < LOD | < LOD | 257.0 | < LOD | 1,599.3 |
| 05.2015 | < LOD | < LOD | < LOD | < LOD | < LOD | < LOD | 987.2 |
| 07.2015 | < LOQ | < LOD | < LOD | < LOD | < LOD | < LOD | 856.0 |
| 08.2015 | < LOD | < LOD | < LOD | < LOD | < LOD | < LOD | 1,043.6 |
| 10.2015 | < LOD | < LOD | < LOD | < LOD | < LOQ | < LOD | 1,133.4 |
| 04.2016 | < LOD | < LOD | < LOD | < LOD | 178 | < LOD | 2,202.0 |
| 06.2016 | < LOD | < LOD | < LOD | < LOD | 150 | < LOD | 2,547.0 |
| 09.2016 | < LOD | < LOQ | < LOD | < LOD | < LOD | < LOD | 1,539.6 |
| 01.2015 | < LOQ | 46.2 | < LOD | < LOD | < LOD | < LOD | < LOQ |
| 05.2015 | < LOQ | < LOQ | < LOD | 1.7 | < LOD | < LOD | < LOQ |
| 07.2015 | 23.1 | 42.3 | < LOD | < LOD | < LOD | < LOD | < LOQ |
| 08.2015 | < LOQ | < LOQ | < LOD | < LOD | 536.0 | < LOD | < LOQ |
| 10.2015 | < LOQ | < LOD | < LOD | < LOD | < LOD | < LOD | 150.0 |
| 04.2016 | < LOD | < LOD | < LOD | < LOD | < LOD | < LOD | < LOQ |
| 06.2016 | < LOD | < LOD | < LOD | < LOD | < LOD | < LOD | 127.1 |
| 09.2016 | < LOD | < LOD | < LOD | < LOD | < LOQ | < LOD | 150.0 |
| 12.2016 | < LOD | < LOD | < LOD | < LOD | < LOQ | < LOD | < LOQ |
| 04.2015 | < LOD | < LOD | < LOD | < LOD | < LOD | < LOQ | 150.0 |
| 06.2015 | < LOQ | < LOQ | < LOD | < LOD | < LOQ | < LOQ | 389.0 |
| 07.2015 | 118.0 | 43.7 | < LOD | < LOD | 738.2 | 48.3 | 957.0 |
| 08.2015 | < LOD | < LOQ | < LOD | < LOD | < LOQ | < LOQ | 846.0 |
| 09.2015 | < LOQ | 26.8 | < LOD | < LOD | 389.5 | < LOQ | 225.0 |
| 10.2015 | 56.7 | 38.7 | < LOD | < LOD | 521.9 | 52.1 | 137.0 |
| 05.2016 | < LOQ | < LOQ | < LOD | < LOD | 200.3 | 300.5 | 206.0 |
| 06.2016 | < LOD | < LOD | < LOD | < LOD | < LOQ | < LOQ | 145.0 |
| 07.2016 | 102.4 | 40.7 | < LOD | < LOD | 450.3 | 46.7 | 667.0 |
| 08.2016 | < LOQ | < LOQ | < LOD | < LOD | < LOQ | < LOQ | 459.0 |
| 04.2015 | < LOD | < LOD | < LOD | < LOD | < LOD | < LOD | < LOD |
| 06.2015 | < LOD | < LOD | < LOD | < LOD | < LOD | < LOD | < LOD |
| 07.2015 | < LOD | < LOD | < LOD | < LOD | < LOD | < LOD | < LOD |
| 08.2015 | < LOD | < LOD | < LOD | < LOD | < LOD | < LOD | < LOD |
| 09.2015 | < LOD | < LOD | < LOD | < LOD | < LOD | < LOD | < LOD |
| 10.2015 | < LOD | < LOD | < LOD | < LOD | < LOD | < LOD | < LOD |
| 05.2016 | < LOD | < LOD | < LOD | < LOD | < LOD | < LOD | < LOD |
| 06.2016 | < LOD | < LOD | < LOD | < LOD | < LOD | < LOD | < LOD |
| 07.2016 | < LOD | < LOD | < LOD | < LOD | < LOD | < LOD | < LOD |
| 08.2016 | < LOD | < LOD | < LOD | < LOD | < LOD | < LOD | < LOD |
| 01.2015 | < LOD | < LOD | < LOD | < LOD | < LOD | < LOD | < LOQ |
| 07.2015 | < LOD | < LOD | < LOD | < LOD | < LOD | < LOD | < LOQ |
| 08.2015 | < LOD | < LOD | < LOD | < LOD | < LOD | < LOD | < LOQ |
| 04.2016 | < LOD | < LOD | < LOD | < LOD | < LOQ | < LOD | < LOQ |
| 06.2016 | < LOD | < LOD | < LOD | < LOD | < LOQ | < LOD | < LOD |
| 08.2016 | < LOD | < LOQ | < LOD | < LOD | < LOQ | < LOD | < LOD |

**PP-LLs**

**MP-LLs**

**RMT-WWs**

**PMT-WWs**

**IN-WWTP**
